# Application of multimodal standardized analgesia under the concept of enhanced recovery after surgery in laparoscopic radical colorectal cancer surgery

**DOI:** 10.3389/fonc.2024.1381809

**Published:** 2024-05-21

**Authors:** Lu Cao, Le Zhang, Baoyu Chen, Likun Yan, Xianpeng Shi, Lifei Tian

**Affiliations:** ^1^ Department of Pharmacy, Shaanxi Provincial People’s Hospital, Xi’an, China; ^2^ Department of Functional Examination, Shaanxi Provincial People’s Hospital, Xi’an, China; ^3^ Department of Anesthesiology, Shaanxi Provincial People’s Hospital, Xi’an, China; ^4^ Department of General Surgery, Shaanxi Provincial People’s Hospital, Xi’an, China

**Keywords:** laparoscopic radical colorectal cancer surgery, multimodal standardized analgesia, enhanced recovery after surgery, ropivacaine, parecoxib

## Abstract

**Aims:**

To observe the efficacy and safety of multimodal standardized analgesia in patients undergoing laparoscopic radical colorectal cancer surgery.

**Methods:**

A prospective, double-blind, randomized study of patients who were admitted to our hospital between December 2020 and March 2022 with a diagnosis of colorectal cancer and who intended to undergo elective laparoscopic radical colorectal cancer surgery was conducted. The participants were randomly divided into two intervention groups, namely, a multimodal standardized analgesia group and a routine analgesia group. In both groups, the visual analogue scale (VAS) pain scores while resting at 6 h, 24 h, 48 h and 72 h and during movement at 24 h, 48 h and 72 h; the number of patient controlled intravenous analgesia (PCIA) pump button presses and postoperative recovery indicators within 3 days after surgery; the interleukin-6 (IL-6) and C-reactive protein (CRP) levels on the 1^st^ and 4^th^ days after surgery; and the incidence of postoperative adverse reactions and complications were recorded.

**Results:**

Compared with the control group, the multimodal standardized analgesia group had significantly lower VAS pain scores at different time points while resting and during movement (*P*<0.05), significantly fewer PCIA pump button presses during the first 3 postoperative days (*P*<0.05), and significantly lower IL-6 and CRP levels on the 1st postoperative day (*P*<0.05). There was no statistically significant difference in the time to out-of-bed activity, the time to first flatus, the IL-6 and CRP levels on the 4th postoperative day or the incidence of postoperative adverse reactions and complications between the two groups (*P >*0.05).

**Conclusion:**

For patients undergoing laparoscopic radical colorectal cancer surgery, multimodal standardized analgesia with ropivacaine combined with parecoxib sodium and a PCIA pump had a better analgesic effect, as it effectively inhibited early postoperative inflammatory reactions and promoted postoperative recovery and did not increase the incidence of adverse reactions and complications. Therefore, it is worthy of widespread clinical practice.

## 1 Introduction

Colorectal cancer is a common malignant tumour of the digestive tract, and its incidence has gradually increased in recent years (1). Laparoscopic radical surgery is the main treatment method (2). Compared with traditional open surgery, laparoscopic colorectal cancer surgery has advantages such as less trauma, faster recovery, and oncological efficacy. In laparoscopic abdominal surgery, small incisions and artificial pneumoperitoneum can still lead to postoperative pain and thus affect the patients’ mood and sleep quality as well as inhibit respiration, leading to complications such as pulmonary atelectasis and lung infections (3). Therefore, good postoperative analgesia is particularly important.

The rapid development of enhanced recovery after surgery (ERAS) concepts (4, 5) has led to an increase in its application in colorectal surgery, and pain management, as a very important concept of ERAS (6), requires standardized analgesia, preventive analgesia, multimodal analgesia and individualized analgesia management for postoperative patients (7). According to the guidelines for minimally invasive colorectal surgery, various analgesic methods are recommended, such as patient controlled intravenous analgesia (PCIA) combined with acetaminophen, nonsteroidal anti-inflammatory drugs (NSAIDs) or opioid agonists, as well as local anaesthetic incisional infiltration, ultrasound-guided transverse abdominis plane block, rectus abdominis muscle sheath block, or other analgesic techniques (7). However, at present, there is a lack of real-world research on the optimal multimodal standardized analgesia method for patients undergoing laparoscopic radical colorectal cancer surgery; such a lack has posed challenges to the clinical operation process (8, 9). Therefore, based on clinical practical needs, this study compared the short-term efficacy of ropivacaine combined with parecoxib sodium with that of a PCIA pump in laparoscopic radical colorectal cancer surgery to determine the best multimodal standardized analgesia method for surgery, with the aim of providing experience and inspiration for clinical practice.

## 2 Materials and methods

### 2.1 Patients

This prospective, double-blind, randomized study was approved by the Ethics Committee of Shaanxi Provincial People’s Hospital. Patients who were admitted to our hospital between December 2020 and March 2022, were diagnosed with colorectal cancer, and who intended to undergo elective laparoscopic radical colorectal cancer surgery were enrolled according to the inclusion and exclusion criteria and then randomized by a computer-generated random allocation sequence into two interventional groups, namely, the multimodal standardized analgesia group and the routine analgesia group. The study was conducted in accordance with the Declaration of Helsinki, and written informed consent was obtained from all the patients.

The inclusion criteria were as follows: (1) aged ≥18 years. (2) diagnosed with colorectal adenocarcinoma by biopsy pathology, with no distant metastasis in relevant auxiliary examinations. (3) American Society of Anaesthesiologists (ASA) grade I ~ III and no history of psychiatric disease. (4) ECOG PS score: 0~1. The exclusion criteria were as follows: (1) required conversion to open approach during the laparoscopic surgery. (2) a history of previous abdominal surgery. (3) a history of allergy to any of the drugs included in the interventions. (4) underwent emergency surgery for intestinal obstruction, perforation or bleeding. (5) underwent intraoperative combined organ resection. (6) were transferred to the intensive care unit for treatment after surgery. (7) a history of drug therapy involving corticosteroids and cyclooxygenase inhibitors within 1 month before surgery. (8) serious diseases such as heart disease (congestive heart failure (NYHA grade II-IV) or coronary artery bypass surgery), liver disease (patients with a serum albumin concentration < 25 g/L or a Child−Pugh score ≥ 10) or kidney disease (patients with a creatinine clearance rate<30 ml/min or with a tendency towards fluid retention). All patients underwent laparoscopic radical colorectal cancer surgery after enrolment, and the surgical procedures were completed in accordance with the “Guidelines for Laparoscopic Colorectal Cancer Radical Surgery (2018 Edition)” (10).

### 2.2 Anaesthesia protocol

After entering the operating room, electrocardiogram, blood pressure and blood oxygen saturation were routinely monitored. General anaesthesia was induced with 1~2 mg of intravenous midazolam, 0.2~0.3 μg/kg of sufentanil, 0.2 mg/kg of cisatracurium and 1~2 mg/kg of propofol. Then, tracheal intubation was performed, and mechanical ventilation was started after successful surgery. Anaesthesia was maintained with 0.1~0.2 mg/kg/minute of sevoflurane. The patients were maintained at a tidal volume of 6~10 ml/kg, respiratory rate of 12 breaths/minute, and end-tidal carbon dioxide of 35~45 mmHg. The same drugs for general anaesthesia induction and maintenance were used for both groups. All surgeries were performed by the same surgeons using the same equipment.

### 2.3 Intervention measures

The patients in the multimodal standardized analgesia group underwent the following before, during and after surgery: (1) preemptive analgesia: 40 mg parecoxib sodium was injected intravenously 30 minutes before anaesthesia induction; and (2) intraoperative analgesia: after closing the aponeurotic layer of the abdominal wall incision, 40 ml 0.5% ropivacaine hydrochloride was used for multipoint infiltration anaesthesia in the aponeurosis layer of each incision and subcutaneous tissue; and (3) postoperative analgesia: after the operation, the anaesthesiologist connected the patient-controlled intravenous analgesia (PCIA) pump to the patient. The formula for PCIA was 100 µg sufentanil + 16 mg ondansetron dissolved in normal saline to 100 mL. The parameter settings for PCIA were as follows: the loading dose was 2 mL, the background infusion dose was 2 mL/h, the single dose was 1 mL, and the lockout time was 20 minutes. Simultaneously, 40 mg parecoxib sodium was injected intravenously every 12 h for the first 3 days after the operation. Ondansetron (4 mg) was injected intravenously if the patient experienced nausea and vomiting. The button on the PCIA pump could be pressed if the pain worsened, and other adverse reactions were treated accordingly.

The patients in the routine analgesia group underwent the following only after surgery: postoperative analgesia: The PCIA was connected by the anaesthesiologist at the end of surgery (with the same formula as above). If the patient developed nausea and vomiting, 4 mg of ondansetron was intravenously injected. The button on the PCIA pump could be pressed when the pain worsened, and 40 mg of parecoxib sodium was injected intravenously for rescue analgesia. Corresponding treatment was administered when other adverse reactions occurred.

### 2.4 Clinical outcomes

According to the relevant published literature and guidelines (7, 11–13), we selected the following items as the clinical outcomes for our article.

1. Pain score: The visual analogue scale (VAS) pain scores while resting at 6 h, 24 h, 48 h and 72 h and during movement at 24 h, 48 h and 72 h (turning over, sitting up and getting out of bed) were recorded in both groups after the operation.
2. The number of PCIA pump button presses and postoperative recovery indicators: The total number of times the patient spontaneously pressed the button on the PCIA pump in the 3-day postoperative period was recorded. The time to out-of-bed activity and the time to first flatus were also recorded.
3. Relevant inflammation markers: Fasting peripheral venous blood specimens were taken from patients in both groups in the early morning of the 1st and 4th postoperative days, and interleukin 6 (IL-6) and C-reactive protein (CRP) levels were detected;
4. Incidence of adverse reactions and complications: The incidences of postoperative nausea and vomiting, skin itching, incision infection, and lung infection were recorded;

### 2.5 Statistical methods

SPSS 23.0 statistical software was used for statistical analysis. The Shapiro−Wilk test was used to test the normality of all variables. Normally distributed continuous variables are expressed as the mean ± standard deviation, and an independent sample *t* test was used for comparisons between the two groups. Nonnormally distributed continuous variables are expressed as medians (interquartile ranges) [M(Q1, Q3)], and the Wilcoxon rank sum test was used. Categorical variables are presented as frequencies and percentages, and the chi-square test or Fisher’s exact test was used for comparisons between the two groups (when the theoretical frequency was less than 1). A *P* value <0.05 indicated a statistically significant difference.

## 3 Results

### 3.1 Clinical characteristics

Ninety-four patients were initially included in this study, and 10 patients were excluded for the following reasons: conversion to open surgery during laparoscopic colorectal cancer surgery (4 patients), taking nonsteroidal anti-inflammatory drugs to control pain within 1 month before surgery (2 patients), transferred to the intensive care unit after surgery (3 patients), and severe renal insufficiency (1 patient). A total of 84 patients were ultimately included in the analysis, with 42 patients in each group. The mean age of patients in multimodal standardized analgesia group was 63.8. ± 11.1 years, including 27 males (64.3%), the mean body mass index (BMI) of patients was 23.3 ± 1.1 kg/m^2^, and the mean operation time was 230.7 ± 49.6 minutes. The mean age of patients in the routine analgesia group was 66.4 ± 13.6 years, including 26 males (61.9%), the mean BMI of patients was 23.4 ± 1.1 kg/m^2^, and the mean operation time was 240.2 ± 54.7 minutes. There was no statistically significant difference in age, sex, body mass index (BMI), ASA classification, surgical time, total incision length, tumour location, or tumour TNM stage between the multimodal standardized analgesia group and the routine analgesia group (*P>*0.05) (**Table 1**).

**Table 1:** Comparison of clinical characteristics between the two groups.

Baseline characteristics	the multimodal standardized analgesia group (n=42)	the routine analgesia group (n=42)	*P-*values
Age/years	63.8. ± 11.1	66.4 ± 13.6	0.338
Male/case (%)	27 (64.3)	26 (61.9)	0.821
BMI (kg/m^2^)	23.3 ± 1.1	23.4 ± 1.1	0.896
Tumor location/case (%)			
Right hemicolon	7 (16.7)	5 (11.9)	0.533
Left hemicolon	3 (7.1)	1 (2.4)	0.608
Sigmoid colon	4 (9.5)	8 (19.0)	0.350
Rectum	28 (66.7)	28 (66.7)	1.000
TNM stages/case (%)			
Stage I	8 (19.0)	10 (23.8)	0.595
Stage II	18 (42.9)	22 (52.4)	0.382
Stage III	16 (38.1)	10 (23.8)	0.157
ASA classification/case (%)			
Classification I	7 (16.7)	8 (19.1)	0.776
Classification II	26 (61.9)	26 (61.8)	1.000
Classification III	9 (21.4)	8 (19.1)	0.786
Surgical time/min	230.7 ± 49.6	240.2 ± 54.7	0.400

### 3.2 VAS pain scores at different time points during the postoperative period

1. Comparison of VAS pain scores at different time points while resting: Compared with those in the routine analgesia group, the VAS pain scores in the multimodal standardized analgesia group were significantly lower at 6 h (*P*=0.024), 24 h (*P*=0.036), 48 h (*P*=0.042) and 72 h (*P*=0.035) after the operation (**Table 2**).
2. Comparison of VAS pain scores at different time points during movement: Compared with the routine analgesia group, the multimodal standardized analgesia group showed a significant decrease in VAS pain scores at 24 hours (*P*<0.001), 48 hours (*P*<0.001), and 72 hours (*P*=0.038) after surgery (**Table 2**).

**Table 2:** Comparison of VAS pain scores between the two groups at different time points during the postoperative period.

Groups	resting				movement		
	6 h	24 h	48 h	72 h	24 h	48 h	72 h
The multimodal standardized analgesia group (n=42)	1.6 ± 0.8	1.5 ± 0.8	1.5 ± 0.7	1.3 ± 0.6	2.3 ± 0.8	1.9 ± 0.8	1.8 ± 0.8
The routine analgesia group (n=42)	3.8 ± 0.9	3.6 ± 0.9	3.0 ± 0.9	1.9 ± 0.7	4.6 ± 1.0	4.0 ± 1.0	3.2 ± 0.7
*P* values	0.024	0.036	0.042	0.035	<0.001	<0.001	0.038

### 3.3 The number of PCIA pump button presses and postoperative recovery indicators

According to **Table 3**, the number of button presses of PCIA in the multimodal standardized analgesia group was significantly less than that in the routine analgesia group (*P*<0.001), but there was no statistically significant difference in the time to first out-of-bed activity (*P*=0.152) or the time to first flatus (*P*=0.423) between the two groups.

**Table 3:** Comparison of the number of PCIA pump button presses and recovery indicators between the two groups.

Groups	the number of PCIA pump button presses (times)	time to first out-of-bed activity (h)	time to first flatus (h)
The multimodal standardized analgesia group (n=42)	1.46 ± 0.57	23.5 ± 4.9	48.1 ± 6.7
The routine analgesia group (n=42)	3.28 ± 0.89	33.3 ± 5.1	54.0 ± 6.4
*P* values	<0.001	0.152	0.423

### 3.4 Relevant postoperative inflammatory indicators

The IL-6 (*P*=0.003) and CRP levels (*P*<0.001) in the multimodal standardized analgesia group were significantly lower than those in the routine analgesia group on postoperative day 1, but the IL-6 (*P*=0.249) and CRP levels (*P*=0.068) on postoperative day 4 were not significantly different (**Table 4**).

**Table 4:** Comparison of relevant postoperative inflammatory indicators between the two groups.

Groups	IL-6 (pg/ml)		CRP (mg/L)	
	postoperative day 1	postoperative day 4	postoperative day 1	postoperative day 4
The multimodal standardized analgesia group (n=42)	36.0 ± 9.0	14.1 ± 4.5	30.8 ± 7.6	16.3 ± 3.7
The routine analgesia group (n=42)	41.8 ± 8.6	18.9 ± 4.4	37.5 ± 7.6	18.9 ± 2.8
*P* values	0.003	0.249	<0.001	0.068

### 3.5 Postoperative adverse effects and complications

In the multimodal standardized analgesia group, there were 5 cases of postoperative nausea, 2 cases of vomiting, 1 case of itchy skin, and 2 cases of incision infection, totalling 10 cases (24.4%). In the routine analgesia group, there were 4 cases of nausea, 1 case of vomiting, 1 case of incision infection, and 2 cases of lung infection, totalling 8 cases (18.6%). There was no statistically significant difference in the incidence of adverse reactions and complications between the two groups (*P* =0.595). See **Table 5**.

**Table 5:** Comparison of postoperative adverse effects and complications between the two groups.

Groups	nausea	vomit	pruritus	incision infection	lung infection	total
The multimodal standardized analgesia group (n=42)	5 (11.9)	2 (4.8)	1 (2.4)	2 (4.8)	0 (0.0)	10 (23.8)
The routine analgesia group (n=42)	4 (9.5)	1 (2.4)	0 (0.0)	1 (2.4)	2 (4.8)	8 (19.0)
*P* values	0.725	0.557	0.314	0.557	0.152	0.595

## 4 Discussion

Laparoscopic radical colorectal cancer surgery, as major cause of injury, can cause the body to release analgesic substances, leading to peripheral sensitization. Moreover, making the surgical incision directly stimulates nociceptive receptors, which can also cause peripheral nerve sensitization, leading to a decrease in the body’s pain threshold, causing significant postoperative pain (14), thus increasing the risk of postoperative complications and affecting early postoperative activities and rehabilitation exercises (7). In addition, if acute postoperative pain is not effectively managed, it may progress to chronic pain that is difficult to control, seriously affecting patient postoperative quality of life.

Contrary to traditional methods, ERAS is a new concept in which a series of optimization measures can be applied during the perioperative period to avoid complications and surgical stress and promote the postoperative recovery of patients (15, 16). Perioperative pain management plays a very important role in the process of ERAS implementation. Multimodal analgesia and standardized analgesia are the core measures for ERAS pain management. Multimodal analgesia refers to the combined application of analgesic drugs with different mechanisms of action and/or multiple analgesic methods that act on different stages and targets of pain to achieve more satisfactory analgesic effects and minimize adverse drug reactions while reducing the impact of pain and drugs on the immune system, cardiovascular system, endocrine system, and nervous system and reducing the occurrence of postoperative complications (17). Standardized analgesia refers to the standardized treatment of perioperative pain, regular recording and evaluation of analgesic effects, timely handling of adverse reactions and various problems that arise during analgesic treatment, and reducing the occurrence of postoperative pain-related complications (7). However, perioperative pain management is receiving increasing amounts of attention. However, relevant studies have shown that the effectiveness of perioperative pain treatment is still unsatisfactory. Two studies in the United States in 2014 showed that 50-70% of patients still experienced moderate-to-severe pain after surgery (18–20). According to a 2017 study investigating the current status of perioperative pain management in 847 hospitals in China by Zhang et al., multimodal analgesia has not been widely popularized for perioperative pain treatment in China, and although postoperative analgesic pumps are widely used in clinical practice, the technical application rate is not high, and a standardized pain management model has not been established (18).

In this study, the VAS score of the multimodal standardized analgesia group was significantly lower than that of the control group, and the number of PCIA pump button presses was significantly lower in the former group, indicating that the multimodal standardized preemptive analgesia method (intravenous injection parecoxib sodium 40 mg at 30 minutes before anaesthesia induction) + intraoperative analgesia (intraoperative ropivacaine for multipoint infiltration anaesthesia) + postoperative analgesia (postoperative administration of parecoxib at regular intervals and PCIA after surgery) had an absolute advantage in terms of analgesic effect compared to the routine analgesia group. First, parecoxib sodium is a new type of nonsteroidal anti-inflammatory drug that selectively inhibits cyclooxygenase-2 (COX-2), which is hydrolysed *in vivo* to valdecoxib, which can inhibit the expression of COX-2 in the periphery and reduce the production of prostaglandins to exert anti-inflammatory and analgesic effects. It can inhibit central COX-2 expression to inhibit pain hypersensitivity, improve the pain threshold and exert an analgesic effect. This product takes effect quickly and has a powerful analgesic effect. Its application before and after surgery can significantly reduce incision pain and visceral pain in laparoscopic colorectal cancer surgery patients (7, 14). Second, as a long-lasting amide-type local anaesthetic, ropivacaine is safe, effective, and has long-lasting effects. At the end of the procedure, infiltration anaesthesia is applied to the myofascial layer of the wound and the subcutis, which provides analgesia by blocking the branch nerves in the myofascial layer of the wound and around the skin. When used in small doses, this product mainly blocks the sensory nerves rather than the motor nerves, effectively alleviating postoperative pain without affecting early ambulation (21).

IL-6 is one of the main proinflammatory factors in the acute phase of the inflammatory response and plays an important role in regulating the body’s response to injury, infection development, etc. CRP is an acute response protein that acts similarly to IL-6 and can reflect the degree of the inflammatory response in the body. The levels of IL-6 and CRP increase rapidly under stressful conditions, such as during surgery, and can affect a patient’s postoperative recovery if they are in a state of high expression after the operation (22). In this study, the IL-6 and CRP levels in the multimodal standardized analgesia group were significantly lower than those in the routine analgesia group on the first postoperative day, possibly because parecoxib sodium can control the inflammatory response of the body at an early stage and reduce the release of inflammatory factors in the postoperative period (23), thus reducing postoperative pain in patients, which is consistent with the findings of current studies (24). In terms of the time to first flatus, although the difference between the two groups was not statistically significant, the time in the former group was still shorter, which may be attributed to the rapid recovery of gastrointestinal function prompted by the shorter time to out-of-bed activity after good analgesia and the increase in the amount of activity.

The overall incidence of adverse reactions and complications, as well as the incidences of nausea, vomiting, skin itching, incision infection and lung infection, were not significantly different between the multimodal standardized analgesia group and the routine analgesia group, indicating that the multimodal standardized analgesia method was safe and feasible and did not increase the incidence of postoperative complications and adverse reactions in patients.

This study has several limitations. (1) This was a single-centre study, and the clinical outcomes might be affected by the medication preferences observed by clinicians to some extent, which might cause some bias in the research results. (2) Only CRP and IL-6 were selected to determine postoperative inflammatory conditions, without considering changes in commonly used clinical inflammatory indicators such as white blood cell count, neutrophil count, and procalcitonin level, which might have an impact on the results. (3) The sample size was small, causing some limitations in the assessment of the impact on clinical outcomes.

## 5 Conclusions

In conclusion, the multimodal standardized analgesia method with ropivacaine combined with parecoxib sodium and a PCIA pump explored in this study has a better analgesic effect on patients undergoing laparoscopic radical colorectal cancer surgery and can effectively inhibit early postoperative inflammatory reactions and promote postoperative recovery without increasing the incidence of adverse reactions and complications. At the same time, the method is simple to apply, easy to master, highly feasible and clinically applicable.

## Data availability statement

The raw data supporting the conclusions of this article will be made available by the authors, without undue reservation.

## Ethics statement

The studies involving humans were approved by Shaanxi Provincial People’s Hospital. The studies were conducted in accordance with the local legislation and institutional requirements. The participants provided their written informed consent to participate in this study.

## Author contributions

LC: Conceptualization, Data curation, Formal analysis, Funding acquisition, Investigation, Methodology, Project administration, Resources, Software, Supervision, Validation, Visualization, Writing – original draft, Writing – review & editing. LZ: Writing – original draft. BC: Writing – original draft. LY: Writing – original draft. XS: Writing – original draft. LT: Conceptualization, Data curation, Formal analysis, Funding acquisition, Investigation, Methodology, Project administration, Resources, Software, Supervision, Validation, Visualization, Writing – original draft, Writing – review & editing.
